# Predictors of surgical outcomes and survival in rectal cancer patients undergoing laparoscopic total mesorectal excision after neoadjuvant chemoradiation therapy: the interest of pelvimetry and restaging magnetic resonance imaging studies

**DOI:** 10.18632/oncotarget.25431

**Published:** 2018-05-18

**Authors:** Nicola de’Angelis, Frederic Pigneur, Aleix Martínez-Pérez, Giulio Cesare Vitali, Filippo Landi, Teresa Torres-Sánchez, Victor Rodrigues, Riccardo Memeo, Giorgio Bianchi, Francesco Brunetti, Eloy Espin, Frederic Ris, Alain Luciani, on behalf of the EuMaRCS Study Group

**Affiliations:** ^1^ Unit of Digestive, Hepato-Pancreato-Biliary Surgery and Liver Transplantation, Henri Mondor Hospital, AP-HP, University of Paris Est, UPEC, Créteil, France; ^2^ Department of Radiology, Henri Mondor Hospital, AP-HP, University of Paris Est, UPEC, Créteil, France; ^3^ Unit of Colorectal Surgery, Department of General and Digestive Surgery, Hospital Universitario Doctor Peset, Valencia, Spain; ^4^ Service of Abdominal Surgery, Geneva University Hospitals and Medical School, Geneva, Switzerland; ^5^ Unit of Colorectal Surgery, Department of General and Digestive Surgery, Hospital Universitario Vall d’Hebron, Barcelona, Spain

**Keywords:** rectal cancer, laparoscopic surgery, total mesorectal excision, magnetic resonance imaging, pelvimetry

## Abstract

**Background:**

Locally advanced rectal cancer (LARC) requires a multimodal therapy tailored to the patient and tumor characteristics. Pretreatment magnetic resonance imaging (MRI) is necessary to stage the primary tumor, while restaging MRI, which is not systematically performed, may be of interest to identify poor responders to neoadjuvant chemoradiation therapy (NCRT), and redefine therapeutic approach. The *EuMaRCS* study group aimed to investigate the role and accuracy of pretreatment (including pelvimetry) and restaging MRIs in predicting surgical difficulties and surgical outcomes in LARC therapy.

**Methods:**

Patients with mid or low LARC who were administered NCRT, who underwent laparoscopic total mesorectal excision, and for whom pretreatment and restaging MRIs were available, were included.

**Results:**

MRIs of 170 patients (median age: 61 years) were reanalyzed by the same radiologist. Pelvimetry differed significantly between males and females, but no gender difference was noted in the clinical and tumor characteristics. Tumor volume and tumor height assessed on the restaging MRI were associated, respectively, with operative time and estimated blood loss. Conversion was predicted by tumor volume, interischial distance and pubic tubercle height. The quality of the surgical resection was found to be a predictor of overall and disease-free survival. The sensitivity and specificity of tumor regression grade 1 to identify a pathologic complete response were 76.9% and 89.3%, respectively.

**Conclusions:**

In LARC management, pelvimetry and restaging MRI may be useful to predict surgical difficulties and surgical outcomes. However, the main independent predictor of patient survival appears to be the achievement of a successful surgical resection.

## INTRODUCTION

Locally advanced rectal cancer (LARC) is associated with poor prognosis and a high risk of developing local and distant metastases [1]. Its management requires the application of a multimodal therapy, which includes neoadjuvant treatments, surgery, and eventually, adjuvant treatments. The cornerstone of the current therapeutic strategies for LARC is to tailor the different available treatments to the patient and tumor characteristics in order to achieve the best tumor response with the minimal associated morbidity [2].

After LARC diagnosis, neoadjuvant chemoradiation therapy (NCRT) is used to decrease the risk of local recurrence and obtain downstaging of the primary tumor before surgery [3]. Complete tumor regression, established as a pathological complete response (pCR; ypT0N0V0), is observed in 10% to 30% of patients [4–6], whereas up to 20% of patients are non-responders [7].

Magnetic resonance imaging (MRI) is nowadays considered the most accurate test to define locoregional clinical staging of rectal cancer [8]. Indications for NCRT in case of LARC should be based on the MRI study performed to stage the primary tumor and assess its resectability (i.e., pretreatment MRI). Moreover, the radiologic measuring of the pelvis, namely, pelvimetry, has been proposed as a helpful tool in determining important predictors of surgical difficulties that may need to be taken into account when planning cancer resection [9–11].

Following NCRT, surgical excision remains the best option to improve disease-free and overall survival in patients with LARC [2, 12, 13]. The oncologic objective is to achieve complete mesorectal excision with clear resection margins [14]. Although it is still under debate [14–16], the use of laparoscopy for rectal cancer has progressively increased with laparoscopic total mesorectal excision (L-TME) and laparoscopic abdominal perineal resection (L-APR) as the most frequently performed procedures in high-volume referral hospitals. Although it improves patients’ prognosis [17], surgery may be also associated with significant morbidity and sequelae, such as fecal incontinence or bladder and sexual dysfunction [18, 19]. Therefore, it has been recently suggested that in selected patients with a clinical complete response to NCRT, a “watch and wait” approach, which avoids surgery in favor of observation, might be justified [20]. Also in this setting, the accuracy of the method for the assessment of tumor response to treatment is of cardinal importance and must differentiate radiation-induced fibrosis or edema from residual cancer in a reliable way. Restaging MRI, performed after the completion of NCRT, has been shown to be acceptably accurate and reproducible in assessing tumor response in some studies but is not routinely performed nor unanimously accepted [21–27].

The *EuMaRCS* (European MRI and Rectal Cancer Surgery) study group aimed to investigate the role of pretreatment and restaging MRI in rectal cancer therapy by analyzing radiological parameters (including pelvimetry) in relation to clinical and histopathological outcomes in order to 1) assess the predictors of surgical difficulties and survival related to the tumor and the patient's characteristics and 2) estimate the diagnostic accuracy of restaging MRI.

## RESULTS

Initially, 184 patients were selected based upon the described inclusion criteria. However, due to the lack of essential MRI sequences or incomplete protocols, which prevented re-evaluation and measurements, 14 patients were excluded. Finally, 170 patients composed the study population.

### Demographic, operative and histopathological variables

Demographic, operative, and histopathological variables are displayed in Table 1. No difference was noted between males and females, except that male patients were more frequently transfused than females (*p* = 0.033). Tumor sterilization (ypT0N0V0) was noted in 25 (22.9%) male and 14 (23%) female patients (*p* = 1). Two male patients (1.17%) presented with ypT0N+. All patients had >2 lymph nodes harvested. A successful resection was obtained in 85 (78%) male and 48 (78.7%) female patients (*p* = 1).

**Table 1:** Demographic, operative and histopathologic variables of patients undergone laparoscopic surgical resection of LARC after NCRT

Variables	Whole sample *n* = 170	Male patients *n* = 109	Female patients *n* = 61	*P* Values
***Demographic and Clinical Variables***				
**Age** (yr) [median (range)]	61 (27–86)	62 (27–86)	59.1 (28.5–86)	0.405
**Male gender** [*n* (%)]	109 (64.1)	-	-	NA
**BMI** (kg/m^2^) [median (range)]	26 (17–46.4)	26 (18.25–38.5)	25.8 (17–46.4)	0.703
**Obesity** (BMI ≥ 30 kg/m^2^) [*n* (%)]	32 (18.8)	19 (17.4)	13 (21.3)	0.539
**ASA score** I/II/III [*n*]	29/86/55	19/50/40	10/36/15	0.209
**Albumin serum level** (g/L) [mean (SD)]	39.29 (5.46)	39.19 (6.18)	39.49 (3.65)	0.695
**Preoperative serum CEA** (U/mL) [mean (SD)]	9.38 (32.07)	11.66 (39.25)	5.07 (6.36)	0.303
**Charlson score** [median (range)]	3 (2–11)	3 (2–11)	3 (2–8)	0.280
**CR possum score** [median (range)]	9 (6–17)	9 (6–15)	9 (6–17)	0.760
**Comorbidity** [*n* (%)]	108 (63.5)	72 (66.1)	36 (59)	0.317
**Comorbidity** >1 [*n* (%)]	62 (36.5)	45 (41.3)	17 (27.9)	0.211
**Diabetes** [*n* (%)]	34 (20)	24 (22)	10 (16.4)	0.266
**Cardiovascular diseases** [*n* (%)]	55 (32.4)	39 (35.8)	16 (26.2)	0.634
**Pulmonary diseases** [*n* (%)]	26 (15.3)	21 (19.3)	5 (8.2)	0.154
**Kidney failure** [*n* (%)]	8 (4.7)	4 (3.7)	4 (6.6)	0.692
**Neurocognitive disorders** [*n* (%)]	5 (2.9)	3 (2.8)	2 (3.3)	0.977
**Previous abdominal surgery** [*n* (%)]	50 (29.4)	27 (24.8)	23 (37.7)	0.206
**Surgical laparoscopic procedures** [*n* (%)]				
- TME with primary anastomosis	136 (80)	85 (78)	51 (83.6)	0.674
- Low Hartmann procedure with TME	13 (7.6)	9 (8.3)	4 (6.6)	
- APR	21 (12.4)	15 (13.8)	6 (9.8)	
***Operative* and *Postoperative Variables***				
**Operative time (min)** [median (range)]	240 (120–550)	235 (130–550)	241.5 (120–550)	0.818
**Conversion to laparotomy** [*n* (%)]	11 (6.5)	10 (9.2)	1 (1.6)	0.100
**Operative blood loss (mL)** [median (range)]	60 (0–2000)	60 (0–2000)	50 (0–1500)	0.775
**Number of transfused patients** [*n* (%)]	13 (7.6)	12 (11)	1 (1.6)	**0.033**
**Time to flatus** [mean (SD)]	2.88 (3.57)	3.01 (4.23)	2.64 (1.81)	0.188
**Return to regular diet** [mean (SD)]	5.45 (5.47)	5.96 (6.38)	4.52 (3.10)	0.915
***ISGRC* anastomotic leakage§ [*n* (%)]**				0.205
- A	3 (2.2)	2 (2.4)	1 (2)	
- B	11 (8.1)	9 (10.6)	2 (3.9)	
- C	8 (5.9)	7 (8.2)	1 (2)	
**Dindo- Clavien classification of postoperative complications** [*n* (%)]				
- I/II	48 (68.6)	34 (64.2)	14 (82.4)	0.353
- III/IV	21 (30)	18 (34)	3 (17.6)	
- V	1 (1.4)	1 (1.9)	0	
**Reoperation** [*n* (%)]	16 (9.4)	13 (11.9)	3 (4.9)	0.303
**Hospital stay, days** [median (range)]	10 (4–70)	10 (4–70)	10 (5–30)	0.968
**Mortality at 90 days** [*n* (%)]	0	0	0	-
**Readmission within 60 days** [*n* (%)]	18 (10.6)	14 (12.8)	4 (6.6)	0.437
**Adjuvant chemotherapy** [*n* (%)]	117 (68.8)	74 (67.9)	43 (70.5)	0.837
***Histopathological (yp) Variables***				
**Tumor largest dimension (cm)** [mean (SD)]	2.38 (1.77)	2.32 (1.54)	2.48 (2.11)	0.719
**Tumor regression grade (MANDARD)** [*n* (%)]				0.668
- I	39 (22.9)	25 (22.9)	14 (23)	
- II	34 (20)	25 (22.9)	9 (14.8)	
- III	53 (31.2)	34 (31.2)	19 (31.1)	
- IV	42 (24.7)	24 (22)	18 (29.5)	
- V	2 (1.2)	1 (0.9)	1 (1.6)	
**ypT stage** [*n* (%)]				0.991
- ypT0	40 (23.5)	26 (23.9)	14 (23)	
- ypT1	10 (5.9)	6 (5.5)	4 (6.6)	
- ypT2	49 (28.8)	31 (28.4)	18 (29.5)	
- ypT3	61 (35.9)	40 (36.7)	21 (34.4)	
- ypT4a	6 (3.5)	4 (3.7)	2 (3.3)	
- ypT4b	4 (2.4)	2 (1.8)	2 (3.3)	
**ypN stage** [*n* (%)]				0.105
- yN0	121 (71.2)	78 (71.6)	43 (70.5)	
- yN1	21 (12.4)	14 (12.8)	7 (11.5)	
- yN1a	10 (5.9)	3 (2.8)	7 (11.5)	
- yN1b	6 (3.5)	4 (3.7)	2 (3.3)	
- yN1c	1 (0.6)	0	1 (1.6)	
- yN2	8 (4.7)	7 (6.4)	1 (1.6)	
- yN2a	3 (1.8)	3 (2.8)	0	
**ypCRM mm** [mean (SD)]	10.01 (8.27)	9.76 (8.04)	10.42 (8.75)	0.797
**Positive ypCRM** [*n* (%)]	16 (9.4)	11 (10.1)	5 (8.2)	0.789
**ypDRM mm** [mean (SD)]	31.58 (24.15)	31.35 (21.69)	31.99 (28.36)	0.757
**Positive ypDRM** [*n* (%)]	6 (3.5)	3 (2.8)	3 (4.9)	0.668
**Complete mesorectal excision** [*n* (%)]	144 (84.7)	92 (84.4)	52 (85.2)	
- ypCRM ≤ 1 mm, ypDRM+	2 (1.4)	1 (1.1)	1 (1.9)	0.864
- ypCRM ≤ 1 mm, ypDRM−	8 (5.6)	5 (5.4)	3 (5.8)	
- ypCRM > 1 mm, ypDRM+	1 (0.7)	1 (1.1)	0	
- ypCRM > 1 mm, ypDRM–	133 (92.4)	85 (92.4)	48 (92.3)	
**Nearly complete mesorectal excision** [*n* (%)]	20 (11.8)	14 (12.8)	6 (9.8)	
- ypCRM ≤ 1 mm, ypDRM+	2 (10)	1 (7.1)	1 (16.7)	0.418
- ypCRM ≤ 1 mm, ypDRM–	3 (15)	3 (21.4)	0	
- ypCRM > 1 mm, ypDRM+	0	0	0	
- CRM > 1 mm, DRM−	15 (75)	10 (71.4)	5 (83.3)	
**Incomplete mesorectal excision** [*n* (%)]	6 (3.5)	3 (2.8)	3 (4.9)	
- ypCRM ≤ 1 mm, ypDRM+	0	0	0	
- ypCRM ≤ 1 mm, ypDRM−	1 (16.7)	1 (33.3)	0	0.368
- ypCRM > 1 mm, ypDRM+	1 (16.7)	0	1 (33.3)	
- ypCRM > 1 mm, ypDRM−	4 (67.7)	2 (66.7)	2 (66.7)	
**Harvested lymph nodes** [mean (SD)]	13.55 (5.93)	13.51 (6.01)	13.62 (5.84)	0.618
**Lymphovascular invasion** [*n* (%)]	19 (11.2)	14 (12.8)	5 (8.2)	0.451
**Perineural invasion** [*n* (%)]	20 (11.8)	11 (10.1)	9 (14.8)	0.457
**Tumor deposit** [*n* (%)]	62 (36.5)	44 (40.4)	18 (29.5)	0.370
**Tumor grade** [*n* (%)]^*^				0.690
- Well differentiated	55 (41.9)	34 (40.5)	21 (44.7)	
- Moderately differentiated	49 (37.4)	30 (35.7)	19 (40.4)	
- Poorly differentiated	27 (20.6)	20 (23.8)	7 (14.9)	

Data are presented for the whole study sample and by gender.

^§^Calculated from laparoscopic TME with primary anastomosis (*n* = 136).

^*^Calculated from non-sterilized tumors (*n* = 131).

BMI stands for body mass index; ASA, for American Society of Anesthesiology; CEA, for carcinoembryonic antigen; TME, for total mesorectal excision; APR, for abdominoperineal resection; T, for tumor stage; N, for node stage; CRM, for circumferential resection margin; and DRM, for distal resection margin.

### Pelvimetry

Measurements on the pretreatment MRI are shown in Table 2. Males and females differed significantly for the majority of the recorded measures of pelvimetry, showing that male patients generally have a narrower and deeper pelvis than female patients.

**Table 2:** Pelvimetry of patients with LARC

Variables	Whole sample *n* = 170	Male patients *n* = 109	Female patients *n* = 61	*P* Values
***Pelvimetry***				
*Transverse measures*				
**Interischiatic spinous distance (mid-pelvic plane)** (mm) [mean (SD)]	97.65 (11.22)	92 (8.26)	107.66 (8.45)	**<0.0001**
**Intertuberous distance (outlet pelvic plane)** (mm) [mean (SD)]	120.11 (16.36)	113.46 (14.65)	131.89 (12.07)	**<0.0001**
**Interacetabular distance (inlet pelvic plane)** (mm) [mean (SD)]	124.68 (8.04)	122.93 (7.64)	127.79 (7.86)	**<0.0001**
*Sagittal measures*				
**Pelvic outlet length (pubic symphysis to the tip of the coccyx distance)** (mm) [mean (SD)]	86.87 (10.91)	85.79 (11.28)	88.78 (10.02)	0.082
**Mid-inlet length (pelvic depth)** (mm) [mean (SD)]	105.61 (11.24)	108.61 (11.34)	100.22 (8.84)	**<0.0001**
**Pelvic inlet length (promontory to pubic symphysis distance)** (mm) [mean (SD)]	109.43 (12.03)	106.82 (10.26)	114.05 (13.55)	**<0.0001**
**Promontory to coccyx distance** (mm) [mean (SD)]	120.37 (15.12)	123.44 (16.04)	114.85 (11.51)	**<0.0001**
**Length of the anterior sacrococcygeal curve** (mm) [mean (SD)]	155.11 (20.19)	157.24 (23)	151.27 (13.09)	**<0.0001**
**S3 (middle third) to promontory distance** (mm) [mean (SD)]	80.02 (7.2)	81.05 (7.65)	78.16 (5.93)	**0.003**
**S3 (middle third) to coccyx distance** (mm) [mean (SD)]	64.64 (9.53)	67.05 (9.5)	60.39 (8.03)	**<0.0001**
**Pubic tubercle height** (mm) [mean (SD)]	52.06 (10.03)	53.60 (11.96)	49.33 (3.79)	**<0.0001**
*Angles*				
**Angle 1 (°)** [mean (SD)]	92.72 (10.11)	92.88 (9.44)	92.44 (11.29)	0.706
**Angle 2 (°)** [mean (SD)]	111.45 (16.82)	111.82 (14.86)	110.79 (19.98)	0.544
**Angle 3 (°)** [mean (SD)]	113.43 (12.35)	111.16 (12.05)	117.47 (11.93)	**0.002**
**Angle 4 (°)** [mean (SD)]	126.19 (7.18)	126.71 (7.29)	125.27 (6.94)	0.225
**Angle 5 (°)** [mean (SD)]	94.71 (6.41)	96.12 (5.77)	92.18 (6.96)	**<0.0001**
**Promontory to the top of the pubic symphysis angle** (°) [mean (SD)]	63.46 (6.77)	63.23 (6.28)	63.87 (7.62)	0.624
**Promontory to the lowest tip of the pubic symphysis angle** (°) [mean (SD)]	39.82 (5.37)	38.92 (4.98)	41.44 (5.71)	**0.003**
*Surface measures*				
S**urface of the sacrum-coccyx concavity** (cm^2^) [mean (SD)]	34.02 (10.37)	35.63 (11.71)	31.11 (6.53)	0.001
**Lesser pelvis surface** (cm^2^) [mean (SD)]	105.76 (13.03)	106.85 (12.55)	103.79 (13.74)	0.179
**Rectal surface at the level of the low-mid-rectum junction** (cm^2^) [mean (SD)]	8.08 (3.61)	8.66 (3.83)	6.94 (2.82)	**0.008**
**Mesorectal surface at the level of the low-mid-rectum junction** (cm^2^) [mean (SD)]	3.95 (1.97)	3.99 (2.04)	3.86 (1.84)	0.644
**Presacral fascia surface at the level of the low-mid-rectum junction** (cm^2^) [mean (SD)]	12.06 (4.66)	12.7 (4.99)	10.8 (3.65)	**0.029**
**Rectal surface at the level of the mid-high rectum junction** (cm^2^) [mean (SD)]	7.45 (4.25)	7.52 (4.36)	7.31 (4.08)	0.854
**Mesorectum surface at the level of the mid-high rectum junction** (cm^2^) [mean (SD)]	19.69 (6.89)	19.63 (5.6)	19.82 (8.93)	0.778
**Presacral fascia surface at the level of the mid-high rectum junction** (cm^2^) [mean (SD)]	27.21 (8.55)	27.25 (7.56)	27.13 (10.28)	0.956
*Derivate measures*				
**Ratio of the pelvic inlet to the pelvic depth** [mean (SD)]	1.04 (0.16)	0.99 (0.13)	1.14 (0.17)	**<0.0001**
**Ratio of the pelvic outlet to angle 2** [mean (SD)]	0.85 (0.7)	0.81 (0.51)	0.91 (0.94)	**0.026**
**Ratio of the pelvic outlet to angle 3** [mean (SD)]	0.77 (0.15)	0.78 (0.16)	0.76 (0.14)	0.692
**Ratio of angle 2 to angle 3** [mean (SD)]	1.12 (0.95)	1.07 (0.79)	1.22 (1.19)	**0.048**

Measures were taken on the pretreatment MRI. Data are presented for the whole study sample and by gender.

### Pretreatment and restaging MRI

The tumor characteristics evaluated on the pretreatment and restaging MRIs are shown in Table 3. No significant differences were noted between males and females. Based on the primary tumor staging on the pretreatment MRI, 84 patients (49.4%) were staged as >cT3b and 146 (85.8%) as cN+. Based on the restaging MRI, 41 patients (24.1%) were staged as >yT3b; 56 (32.9%), as yN+. A significant decrease in the tumors’ largest dimension (*p <* 0.0001) and volume (*p* < 0.0001) was observed between pretreatment and restaging MRI assessment, without differences between genders (*p* = 0.262 and *p* = 0.661, respectively).

**Table 3:** Pretreatment MRI and restaging MRI characteristics of patients with LARC undergone laparoscopic resection after NCRT

Variables	Whole sample *n* = 170	Male patients *n* = 109	Female patients *n* = 61	*P* Values
***Pretreatment MRI measurements***				
**Tumor largest dimension** (mm) [mean (SD)]	49.57 (17.44)	49.80 (18.11)	49.17 (16.32)	0.891
**Tumor circumferential extension** [*n* (%)]				0.119
**- 25%**	9 (5.3)	7 (6.4)	2 (3.3)	
**- 50%**	52 (30.6)	30 (27.5)	22 (36.1)	
**- 75%**	53 (31.2)	30 (27.5)	23 (37.7)	
**- 100%**	56 (32.9)	40 (38.5)	14 (23)	
**Tumor height** (mm) [mean (SD)]	49.14 (17.99)	49.42 (18.7)	48.64 (16.8)	0.811
**DRM** (mm) [mean (SD)]	39.49 (28.41)	38.86 (27.58)	40.59 (30.02)	0.739
**cT stage** [*n* (%)]				0.672
- cT1	0	0	0	
- cT2	15 (8.8)	8 (7.3)	7 (11.5)	
- cT3a	17 (10)	12 (11)	5 (8.2)	
- cT3b	54 (31.8)	33 (30.3)	21 (34.4)	
- cT3c	33 (39.4)	25 (22.9)	8 (13.1)	
- cT3d	15 (8.8)	8 (7.3)	7 (11.5)	
- cT4a	23 (13.5)	15 (13.8)	8 (13.1)	
- cT4b	13 (7.6)	8 (7.3)	5 (8.2)	
**cN stage** [*n* (%)]				0.699
- cN0	24 (14.1)	17 (15.6)	7 (11.5)	
- cN1	97 (57.1)	60 (55)	37 (60.7)	
- cN2	49 (28.8)	32 (29.4)	17 (27.9)	
**CRM** (mm) [mean (SD)]	10.89 (9.33)	11.52 (9.98)	9.71 (7.96)	0.377
**Maximum tumor thickness** (mm)[mean (SD)]	15.72 (9.05)	15.47 (8.93)	16.16 (9.31)	0.873
**EMVI positive** [*n* (%)]	64 (37.6)	47 (43.1)	17 (27.91)	0.144
**Tumor volume** (cm^2^) [mean (SD)]	29.28 (37.93)	28.46 (36.13)	30.81 (41.4)	0.617
***Restaging MRI measurements (ymr)***				
**ymrTumor largest dimension** (mm) [mean (SD)]	29.88 (15.83)	30.72 (15.54)	28.4 (16.35)	0.543
**ymrTumor circumferential extension (n%)**				0.417
**- 0%**	31 (18.2)	16 (14.7)	15 (24.6)	
**- 25%**	13 (7.6)	8 (7.3)	5 (8.2)	
**- 50%**	68 (40)	44 (40.4)	24 (39.3)	
**- 75%**	29 (17.1)	19 (17.4)	10 (16.4)	
**- 100%**	29 (17.1)	22 (20.2)	7 (11.5)	
ymrTumor height	28.48 (15.99)	28.81 (15.18)	27.91 (17.45)	0.855
**ymrDRM** (mm) [mean (SD)]	41.94 (28.5)	41.92 (28.34)	41.93 (29.07)	0.903
**ymrT stage** [*n* (%)]				0.300
- ymrT0	31 (18.2)	18 (16.5)	13 (21.3)	
- ymrT1	2 (1.2)	2 (1.8)	0	
- ymrT2	59 (34.7)	38 (34.9)	21 (34.4)	
- ymrT3	1 (0.6)	1 (0.9)	0	
- ymrT3a	7 (4.1)	6 (5.5)	1 (1.6)	
- ymrT3b	29 (17.1)	17 (15.6)	12 (19.7)	
- ymrT3c	19 (11.2)	10 (9.2)	9 (14.8)	
- ymrT3d	7 (4.1)	6 (5.5)	1 (1.6)	
- ymrT4a	10 (5.9)	9 (8.3)	1 (1.6)	
- ymrT4b	5 (2.9)	2 (1.8)	3 (4.9)	
**ymrN stage** [*n* (%)]				0.295
- ymrN0	114 (67.1)	71 (65.1)	43 (70.5)	
- ymrN1	52 (30.6)	34 (31.2)	18 (29.5)	
- ymrN2	4 (2.4)	4 (3.7)	0	
**ymrCMR status >2 mm** [*n* (%)]	101 (78.9)	67 (79.8)	34 (77.3)	0.821
ymrTRG score				0.198
- 1	44 (25.9)	27 (24.8)	17 (27.9)	
- 2	45 (26.5)	35 (32.1)	10 (16.4)	
- 3	43 (25.3)	27 (24.8)	16 (26.2)	
- 4	36 (21.2)	19 (17.4)	17 (27.9)	
- 5	2 (1.2)	1 (0.9)	1 (1.6)	
**ymrMaximum tumor thickness** (mm) [mean (SD)]	9.27 (6.50)	9.67 (6.44)	8.58 (6.62)	0.216
**ymrEMVI positive** [*n* (%)]	45 (26.5)	34 (31.2)	11 (18)	0.071
**ymrTumor volume** (cm^2^) [mean (SD)]	10.65 (20.55)	10.49 (19.58)	10.94 (22.47)	0.542

Data are presented for the whole study sample and by gender.

DRM stands for distal resection margin; CRM, for circumferential resection margin; EMVI, for extramural vascular invasion; and TRG, for tumor regression grade; (ymr) identifies the restaging MRI assessments.

### Multivariate analysis outcomes

Univariate and then multivariate analyses were run to identify factors associated with indicators of surgical difficulties and surgical outcomes (Table 4). Gender was not associated with any of these indicators. Conversely, the tumor volume and the tumor height assessed on the restaging MRI were associated, respectively, with the operative time and the estimated blood loss. Conversion was predicted by tumor volume, interischial distance, and pubic tubercle height. A ymrCRM <2 mm was found to be a associated with postoperative complications and successful resection.

**Table 4:** Multivariate analysis of the association between pelvimetry and MRI characteristics with indicators of operative difficulties and postoperative outcomes on the whole study population (*n* = 170)

Outcomes	Predictive Factor	β Coefficient	*t*	*P* Value
**Operative time**	**ymrTumor volume** *(restaging MRI)*	0.247	2.40	**0.018**
**Estimated blood loss**	**ymrTumor height** *(restaging MRI)*	0.307	3.01	**0.003**
**Outcomes**	**Predictive Factor**	**OR**	**95% CI**	***P* Value**
**Conversion**	**Interischiatic spinous distance**	0.85	0.74–0.97	**0.018**
	**Pubic tubercle height**	1.28	1.01–1.61	**0.042**
	**ymrTumor volume** *(restaging MRI)*	1.02	1.01–1.04	**0.005**
**Postoperative complications**	**Intertuberous distance**	0.97	0.93–0.99	**0.014**
	**Angle 1**	0.94	0.89–0.98	**0.009**
	**ymrCRM < 2 mm** *(restaging MRI)*	3.57	1.38–0.09	**0.008**
**Successful resection**	**ymrCRM < 2 mm** *(restaging MRI)*	0.22	0.08–0.59	**0.003**

Only significant predictors are shown.

CRM stands for circumferential resection margin; OR, for odds ratio; and CI, for confidence interval.

### Survival outcomes

**Figure 1:**
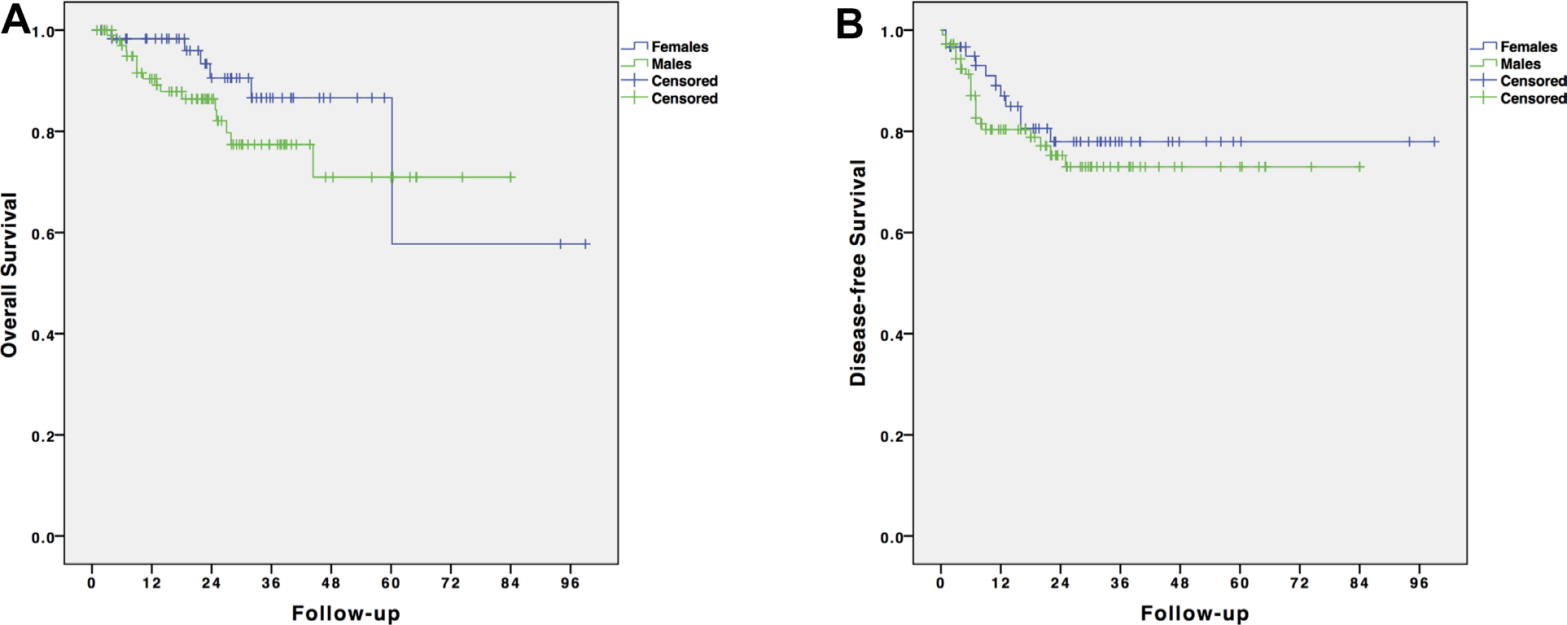
Survival analysis of patients with LARC undergone laparoscopic resection after NCRT (**A**) Overall survival by gender; (**B**) Disease-free survival by gender.

The mean overall follow-up time was 25.01 months (SD: 18.53 months). The overall survival (OS) and disease-free survival (DFS) curves are shown in Figure 1. The 1-, 2- and 3-year OS rates were, respectively, 98%, 91.5% and 89.1% for the male group and 96.3%, 90.5% and 87% for the female group (*p* = 0.164). The mean OS for the male group was 66.12 months (SD: 4.14 months) and that for the female group was 77.53 months (SD: 9.83). The 1-, 2- and 3-year DFS rates were, respectively, 79%, 73.1% and 68.9% for the male group and 87%, 75.2% and 69.4% for the female group (*p* = 0.439). Disease recurrence over the entire follow-up period was observed in 34 patients (20.1%); 23 (21.1%) were males, and 11 (18%) were females (*p* = 0.693). Overall, 6 patients (17.6%) had local recurrence, and 28 patients (82.4%) had distant metastases (including 6 patients who experienced both local and distant recurrence). Distant recurrence included isolated liver metastases (*n* = 7), isolated pulmonary metastases (*n* = 11), liver and pulmonary metastases (*n* = 5), carcinosis (*n* = 3), and systemic metastatic disease (*n* = 2). No gender difference was found (*p* = 0.110). Predictors of OS and DFS are shown in Table 5. The only significant predictor of OS was the quality of the surgical resection, whereas the stage ymr>T3b, the achievement of a successful resection, the ypN+ status, and the ypT4 stage were significant predictors of DFS.

**Table 5:** Univariate and multivariate Cox regression hazard analyses for predictors of overall and disease-free survival in the whole study sample

	Whole study sample (*N* = 169^*^)							
	Overall survival				Disease-free survival			
Variables	Univariate analysis		Multivariate analysis		Univariate analysis		Multivariate analysis	
	HR (95% CI)	*P* Value	Adjusted HR (95% CI)	*P* Value	HR (95% CI)	*P* Value	Adjusted HR (95% CI)	*P* Value
**Gender: male vs. female**	0.52 (0.20–1.32)	0.171			0.75 (0.37–1.55)	0.443		
**Comorbidity ≥1**	1.17 (0.51–2.71)	0.710			1 (0.49–2.03)	0.990		
**L-APR vs. L-TME**	0.98 (0.29–3.32)	0.983			0.67 (0.27–1.62)	0.375		
**Tumor height <4 cm** *(pretreatment MRI)*	0.96 (0.39–2.37)	0.943			1.21 (0.57–2.6)	0.617		
**CRM <2 mm**	1.24 (0.48–3.23)	0.649			**4.14** **(2.08–8.23)**	**<0.0001**		
**ymrCRM <2 mm**	1.09 (0.40–2.97)	0.854			**4.14** **(2.10–8.14)**	**<0.0001**		
**Tumor largest dimension**	1.01 (0.99–1.04)	0.133			**1.02** **(1.1–1.04)**	**0.006**		
**ymrTumor largest dimension**	1.02 (0.99–1.04)	0.076			**1.03** **(1.01–1.04)**	**<0.0001**		
**ymrN+ vs. ymrN0**	1.20 (0.53–2.73)	0.662			**2.82** **(1.53–5.22)**	**0.001**		
**ymr>T3b stage**	**2.64** **(1.15–6.08)**	**0.022**			**7.31** **(3.60–14.80)**	**<0.0001**	**2.50** **(1.09–5.70)**	**0.029**
**Intraoperative transfusion**	1.81 (0.61–5.43)	0.285			1.54 (0.37–6.42)	0.554		
**Conversion to open surgery**	1.27 (0.29–5.41)	0.749			**3.72** **(1.53–9)**	**0.004**		
**Postoperative complication**	1.53 (0.67–3.49)	0.306			1.13 (0.56–2.25)	0.729		
**Unsuccessful vs. successful surgical resection**	**3.28** **(1.44–7.45)**	**0.004**	**3.89** **(1.64–9.22)**	**0.002**	**3.45** **(1.75–6.79)**	**<0.0001**	**2.22** **(1.03–4.79)**	**0.042**
**R1 vs. R0 resection**	**2.83** **(1.25–6.44)**	**0.013**			**3.94** **(2.15–7.24)**	**<0.0001**		
**ypN+ vs. ypN0**	1.97 (0.83–4.64)	0.120			**4.71** **(2.35–9.43)**	**<0.0001**	**2.70** **(1.27–5.74)**	**0.010**
**ypT4 vs. ypT1 to 3**	**4.47** **(1.48–13.46)**	**0.008**			**19.35** **(8.46–44.26)**	**<0.0001**	**4.58** **(1.72–12.19)**	**0.002**
**Harvested lymph nodes <12**	0.98 (0.41–2.31)	0.963			0.79 (0.38–1.66)	0.542		
**Poor differentiation**	2.05 (0.72–5.75)	0.175			0.58 (0.27–1.25)	0.164		
**ymrTRG 4-5 vs. 1 to 3**	1.74 (0.73–4.12)	0.204			**4.09** **(2.08–8.02)**	**<0.0001**		

L-TME stands for laparoscopic total mesorectal excision; APR, for abdominoperineal resection; CRM, for circumferential resection margin; T, for tumor stage; N, for node stage; HR, for hazards ratio; and CI, for confidence interval.

HR <1 indicates an improvement in survival (positive prognostic factor); HR >1 indicates worse survival (negative prognostic factor).

Significant *p* values are in bold characters.

^*^After removing patients deceased during the postoperative period (*n* = 1).

### Concordance between histopathologic and restaging MRI assessments

The PPVs of ymrT staging to correctly identify the ypT0 stage, ypT1/T2 stages, and ypT3/T4 stages were, respectively, 83%, 55.7%, and 68.8%. The PPVs of ymrN staging to correctly identify the ypN0 stage and ypN+ stages were, respectively, 87.7%, and 62.5%. Positive EMVI on the surgical specimens was found in 19/170 patients. The sensitivity, specificity, PPV, and NPV of ymrEMVI were, respectively, 68.4%, 78.8%, 28.8% and 95.2%. The pCR rate (ypT0N0V0) of the specimens was 39/170 patients. The sensitivity and specificity of ymrTRG 1 to 3 to identify pCR were 100% and 29%, respectively, with a false-positive rate of 70.9%. The sensitivity and specificity of ymrTRG 1 to identify pCR were 76.9% and 89.3%, respectively, with a false-positive rate of 10.7%. The PPV and NPV were 68.2% and 92.8%, respectively.

## DISCUSSION

By analyzing pretreatment and restaging MRI characteristics together with the clinical and histopathological outcomes, the present results suggest that pelvimetry and restaging MRI are of interest to predict surgical difficulties and surgical outcomes (i.e., successful resection) as well as to identify pCR to NCRT, which is necessary to tailor treatment strategies in LARC patients. However, the multivariate analyses support that the main independent predictor of OS and DFS in patients with mid- and low rectal cancer is the achievement of complete mesorectal excision with clear resection margins.

The usefulness of measuring the pelvic bony dimensions by pelvimetry on MRI remains debated, although several studies have correlated certain pelvis characteristics with indicators of surgical difficulties, such as operative time and blood loss [9–11, 28–31]. In general, surgery is usually easier in females who present with a shallower and larger pelvis [11, 32]. Ferko *et al.* [29] found that the pelvic entrance dimension was correlated with the quality of the TME. Killeen *et al.* [10] reported that only pelvic outlet measurements (e.g., angles 2 and 3) were independently associated with operative time, without differences between sex, age, and CRM status. Salerno *et al.* [11, 30] demonstrated an important overlap between male and female pelvimetry, with the most significant gender-related differences at the level of the transverse mid-inlet and pelvic outlet diameter [11]. However, the authors concluded that CRM positivity cannot be predicted by pelvimetry in patients with rectal cancer, expect for tumor height [30]. More recently, Escal *et al.* [31] identified the mesorectal fat area and the intertuberous distance as the most predictive measurements, which were included together with the need for coloanal anastomosis and the patient BMI in a predictive score to grade the risk of surgical difficulty. The present study confirmed that male and female patients are substantially different in their pelvis anatomy and mesorectal package [11, 33] and that few transverse pelvic dimensions are indeed predictors of surgical difficulties. It is noteworthy that most of the previously published studies considered heterogeneous samples of patients with different tumor locations, treatment protocols (e.g., with or without CRT), and surgical approaches [9–11, 28, 29, 31, 34]. This may bias the association between the bony structures assessed by pelvimetry and the surgical outcomes if the tumor characteristics, especially in the restaging MRI, are not taken into account. To the best of our knowledge, this is the first time that pelvimetric parameters are considered together with the characteristics of the surrounding soft tissues and organs, as well as the tumor features assessed on both the pretreatment and the restaging MRIs in a homogenous sample of patients with only mid- or low rectal cancer who underwent the same therapeutic protocol including NCRT and laparoscopic total mesorectal excision. The results show that, among all considered variables, only the transverse measure of the pelvis mid-plane and the pelvis deepness are related to the risk of conversion from laparoscopy to open surgery, with a larger interischiatic spinous distance and a shallow pubic tubercle height associated with a lower risk of conversion. Similarly, a larger intertuberous distance (pelvis outlet) and a larger angle between the superior anterior pubis, the sacrovertebral angle, and the mid-S3 (angle 1) are associated with a lower risk of postoperative complications. These findings support the interest in assessing transverse measures at the level of the pelvis mid-plane and outlet to identify unfavorable anatomies, which must always be related to the tumor characteristics [9, 10]. Indeed, the difficulties in the tumor resection are potentially influenced by the delimiting bony structures, but the chance to obtain a successful resection seems to be independent of the anatomic features rather being determined by the tumor characteristics and, most importantly, by the CRM.

Other predictors of surgical difficulties were the tumor volume, the tumor height, and the ymrCRM<2 mm, all assessed on the restaging MRI. Precisely, greater ymrTumor volumes were significantly associated with a longer operative time and higher risk of conversion, whereas a greater tumor height was associated with increased blood loss. A ymrCRM<2 mm was a risk factor for the occurrence of postoperative complications and reduced the chance of obtaining a successful resection.

Previous studies reported an overall high accuracy of restaging MRI in excluding CRM involvement [27], which is known to be a significant predictor of distant and local recurrence [35, 36]. In this study, CRM assessment on the restaging MRI was the only significant predictor of achieving a successful or unsuccessful resection. Moreover, a CRM<2 mm, assessed on both pretreatment and restaging MRI, was found to be a predictor of DFS, although it was no more significant when analyzed in the regression model. Conversely, an ymrT stage greater than T3b, an ypT4 stage and an ypN+, together with the successful resection, remained significantly associated with DFS in the multivariate analysis. For the OS, only the achievement of a successful resection was a significant predictor, with an almost four-fold increased risk of worsened survival for patients experiencing unsuccessful resection. These results confirmed the role of T and N staging as prognostic factors [37–39]; pathologic T4 stage and N+ stages are known to be associated with worse survival [12], but interestingly, an ymrT stage greater than T3b also resulted as a significant predictor of DFS in patients with LARC, supporting that advanced stage tumors and poor responders are more vulnerable to local and distance recurrence [39–41]. In addition, the present results stress the importance of achieving complete mesorectal excision with clear resection margins to improve patient's OS and DFS rates [14, 42]. Once again, it appears that surgery has a cardinal role in the management of LARC, and the oncological adequacy of the surgical resection has a drastic impact on survival independently from the T and N stages or R resection.

Restaging MRI has been proposed as a tool to identify poor responders to NCRT, optimize systemic treatment before rectal cancer resection, and estimate surgical difficulties and outcomes [27, 43]. Thus, restaging MRI may have an essential role in tailoring the therapeutic approach to the specific patient and tumor characteristics, which imply the adoption of the best surgical techniques, including laparoscopic [14, 17, 19], robotic [44, 45] or transanal surgery [46, 47], as well as the best adjuvant treatment, if needed. However, based on a recent meta-analysis, restaging MRI was considered not accurate enough for clinical application and highly variable depending on the different T stages [27]. The present study suggests that restaging MRI has a good PPV for the detection of T0 stages but the PPV drastically decreases for T stages ≥1, confirming that the accuracy of MRI for restaging LARC varies depending on the different T stages [22, 25, 27]. The assessment of ymrEMVI shows acceptable sensitivity and specificity, but a patient with a positive ymrEMVI has only a 28.8% chance of actually having vascular invasion. As known, the PPV is influenced by the sensitivity of the test and the prevalence of the disease in the analyzed population; in this case, only 19/170 patients (11.17%) had a positive EMVI, thus limiting the observed PPV compared to other studies in which the accuracy of ymrEMVI was higher [24, 48, 49]. Using ymrTRG to identify patients with a pCR would be of cardinal importance in the choice of the therapeutic approach. Bhoday *et al.* [22] suggested that ymrTRG 1 to 3 were the best ways of searching for patients who are suitable for deferral of surgery. In the present study, 100% sensitivity in detecting pCR was found by considering ymrTRG 1 to 3 but with a very low specificity and a high false-positive rate that limited the accuracy of restaging MRI. Indeed, to reliably and accurately identify complete responders to NCRT that may be good candidates for the “watch and wait” approach, restaging MRI should be associated with adequate sensitivity, specificity, PPV and NPV. A better balance between these parameters is found by considering only ymrTRG 1, which is associated with 76.9% of sensitivity, 89.3% of specificity, 68.2% of PPVs, and 92.8% of NPVs. In other words, an ymrTRG 1 in the restaging MRI will identify almost all patients with a pCR at the time of surgery, with a 10.7% false-positive rate. However, the threshold of the false-positive rate that can be considered acceptable in clinical practice to opt for deferral of surgery remains to be established, leaving the role of restaging MRI or other promising diagnostic tools (e.g., PET-MRI) to be further assessed in future studies.

The present study has strengths and limitations. It considered a relatively large sample of patients presenting with homogenous clinical and histopathological characteristics that were treated with the same therapeutic protocol for locally advanced mid and low rectal cancer. The available pretreatment and restaging MRIs were reevaluated by only one highly experienced radiologist using standardized measurements, which increased the reliability of the analyzed data [11, 21, 22, 24, 39]. However, as a retrospective multicentric study involving 4 high-volume referral hospitals, the results should be interpreted by taking into account potential selection bias and limited external validity.

In conclusion, in the management of locally advanced rectal cancer, successful surgical resection including complete mesorectal excision and clear resection margins is the main independent predictor of OS and DFS. Thus, any attempt should be made to achieve successful tumor resection. Pelvimetry and restaging MRI may be useful to predict surgical difficulties and surgical outcomes. In particular, restaging MRI can be used to identify responders to NCRT and optimize treatment by a tailored therapy, which may include different surgical strategies up to a conservative “watch and wait” approach.

## MATERIALS AND METHODS

### Study population

The EuMaRCS is a multicenter study group involving four European referral hospitals: Henri Mondor University Hospital of Créteil, France; Doctor Peset University Hospital of Valencia, Spain; Geneva University Hospital of Geneva, Switzerland; and Vall d’Hebron University Hospital of Barcelona, Spain. All participating centers contributed to build a database of patients diagnosed with LARC who underwent MRI before and after NCRT between January 2010 and January 2016.

To be included in the study, patients should have been diagnosed with histologically proven, locally advanced (American Joint Committee on Cancer (AJCC) stages I to IIIc) [50] mid or low rectal cancer (up to 12 cm from the anal verge); they should have completed a long-course NCRT with a total radiation dose of 45–50.4 Gy delivered in daily fractions of 1.8–2 Gy over a 5- to 6-week period combined with 5-fluorouracil or capecitabine (Xeloda) [51]; they should have undergone MRI including diffusion-weighted magnetic resonance imaging (DWI) before (pretreatment MRI) and after (restaging MRI) NCRT [21]; and they should have been operated on by elective ‘‘up-to-down’’ laparoscopic anterior resection (LAR) with total mesorectal excision (L-TME) [5, 52] or laparoscopic abdominoperineal resection (L-APR) [53, 54]. L-APR with colostomy construction was performed for cancer infiltrating the anal sphincter not separable from the external sphincter muscles [12]. Conversion was defined by the need for midline laparotomy to complete the operation [55]. All procedures were carried out by senior colorectal surgeons experienced in minimally invasive surgery.

After the treatments, the patients were followed every 3 months for the first 2 years and every 6 months thereafter. Blood tests with biomarkers were requested at each visit; full colonoscopy was routinely performed 1–2 years after surgery and then once every 4 years. If tumor recurrence was suspected, MRI and/or PET-CT were performed to confirm the diagnosis.

The study was conducted in accordance with the ethical principles described in the Declaration of Helsinki.

### Study outcomes

The following patient- and tumor-related variables were collected and analyzed: demographic and clinical characteristics, MRI and NCRT data, operative and postoperative outcomes (including postoperative complications classified by Dindo-Clavien [56] and anastomotic leakage classified by the International Study Group of Rectal Cancer criteria [57]), tumor recurrence rate, overall survival (OS) and disease-free survival (DFS) rates, and pathologic assessment. The pathologic assessment (yp) included the quality of the mesorectum (i.e., complete, nearly complete, or incomplete [58]), the radicality of surgery (i.e., R0), and the number of harvested lymph nodes. The involvement of the circumferential resection margins (ypCRM) was defined as ≤1 mm distance between the deepest cancer invasion and the surgical resection margin [15, 58], and the involvement of the distal resection margins (ypDRMs) was defined as ≤1 mm between the closest tumor to the cut edge of the tissue [15]. Surgical oncologic success was defined as meeting all the following criteria: i) clear ypCRM, ii) clear ypDRM, and iii) complete mesorectal excision quality [14–16]. The tumor regression grade (TRG) was classified according to the 5-point scale of Mandard [59]. A pathologic complete response (pCR) was defined as the absence of gross and microscopic tumor cells in the specimen in accordance with the nodal status, and in the vasculature beyond the muscularis propria (ypT0N0V0) [5, 22, 24]. When only acellular mucinous bands were observed, the response was considered complete [60].

### Imaging evaluation

Pretreatment MRI was used for primary tumor staging; restaging MRI was performed 6 to 8 weeks after completion of NCRT and was used for tumor restaging and tumor response evaluation. All MRIs were performed according to standard protocols (2-dimensional T2-weighted fast spin-echo sequences in 3 orthogonal directions and an additional diffusion-weighted sequence in the axial plane) [21]. All MRI data were reanalyzed and measured by one radiologist (FP) highly experienced in MRI studies and blinded to the patients’ clinical and histopathological information. The intra-examiner agreement was tested before initiating MRI re-evaluations and showed an intra-class correlation coefficient >0.9.

**Figure 2:**
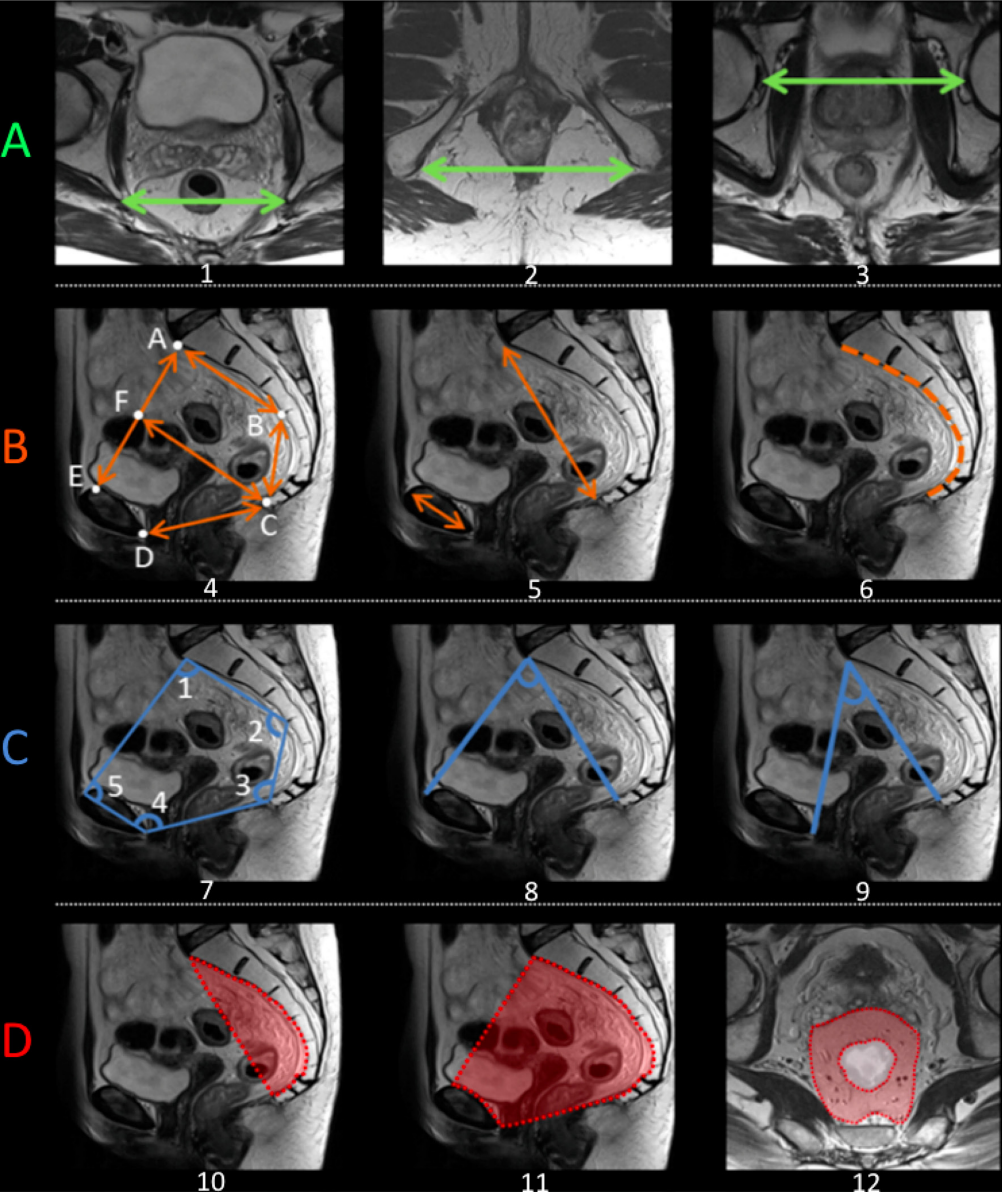
Schematic representation of the main pelvimetric measurement on the pretreatment MRI (**A**) Transverse measures: 1) Interichiatic spinous distance; 2) Intertuberous distance; 3) Interacetabular distance. (**B**) Sagittal measures: 4) AB: S3 to promontory distance; BC: S3 to coccyx distance; CD: Pubic symphysis to the tip of the coccyx distance (pelvic outlet length); AE: Promontory to pubic symphysis distance (pelvic inlet length); CF: mid-inlet length (pelvic depth); 5) Promontory to coccyx distance and pubic tubercle height; 6) Length of anterior sacro-coccigeal curve. (**C**) Angles: 7) Angle 1: superior anterior pubis – sacrovertebral angle – mid-S3; Angle 2: sacrovertebral angle – mid-S3 – coccyx; Angle 3: mid-S3 – coccyx – inferoposterior pubis; Angle 4: coccyx – inferoposterior pubis – superior anterior pubis; Angle 5: inferoposterior pubis – superior anterior pubis – sacrovertebral angle. 8) Promontory to the top of the pubic symphysis angle; 9) Promontory to the lowest tip of the public symphysis angle. (**D**) Surface measures: 10) Surface of the sacrum-coccyx concavity; 11) Lesser pelvis surface; 12) Mesorectal surface at the level of mid-high rectum junction. MRIs were performed according to standard protocols with an external surface coil (on a 1,5T or 3T MRI system). All protocols should have at least 3DT2 weighted or 2DT2 weighted sequences in 3 planes. The axial T2-weighted sequences were angled perpendicular to the tumor axis as defined on sagittal T2-weighted images. Slice thickness for axial sequences should be ≤3 mm. A diffusion-weighted sequence including at least a *b*-value of ≥800 should be included in the restaging MRI protocol.

Pelvimetry was realized on the pretreatment MRI according to previously published criteria providing transverse, sagittal, angles, and surface measures [10, 11, 28–31, 61]. This approach produced 26 individual pelvimetric values describing the width, depth and angles of the lesser pelvis for each patient [29] (Figure 2). The surface was calculated in cm^2^ between different reference points of the pelvis [31]; the rectal surface and the mesorectal surface (horizontal plane) were evaluated at the level of the junction between the high and mid-rectum and the mid- and low rectum. Surface measures were made perpendicular to the rectum axis. In addition, 4 derivate measures were taken [10, 11].

On the pretreatment MRI, cT stage, cN stage and extramural venous invasion (EMVI) status were assessed according to previously published criteria [39, 62]. On the restaging MRI, the degree of response to the NCRT (ranging from 1 to 5) was evaluated based on the magnetic resonance tumor regression grade (mrTRG) score [63, 64], with a mrTRG of 4 or 5 identifying poor responders [22]. The mrEMVI was assessed as described by Chand *et al.* [24]. Tumor volume was assessed on both pretreatment and restaging MRI as described by Lambregts *et al.* [21].

### Statistical analysis

To investigate eventual gender-related differences in pelvimetry measurements and surgical outcomes, males and females were compared by using chi-square test, Fisher's exact test, or Mann–Whitney *U* test. Differences between pretreatment and restaging MRI were assessed by using repeated measures ANOVA. Binary, multimodal, or linear regression analyses (backward models) were performed to assess predictors of surgical difficulties (i.e., estimated based on operative time, blood loss, and conversion rate) [31, 65, 66] and surgical success (i.e., postoperative complications and successful resection rate) [14] by including in the multivariate analysis all variables that reached a *p* value < 0.2 in the univariate analysis. The OS and DFS rates at 1, 2 and 3 years were analyzed using the Kaplan–Meier method and compared between groups using the log rank (Mantel–Cox) test. Potential prognostic factors of survival were evaluated by Cox proportional hazards models. Sensitivity, specificity, positive predictive value (PPV), and negative predictive value (NPV) were calculated between the histopathological assessment and the restaging MRI parameters. Statistics were performed using SPSS (Statistical Package for Social Science, IBM SPSS Statistics, Version 23 for Macintosh; IBM Corp., Armonk, NY, USA). A *p* value < 0.05 was considered statistically significant.

## Abbreviations

AJCC: American Joint Committee on Cancer
ypCRM: Circumferential resection margins
DFS: Disease-free survival
DRMs: Distal resection margins
DWI: Diffusion-weighted magnetic resonance imaging
*EuMaRCS*: European MRI and Rectal Cancer Surgery
EMVI: Extramural venous invasion
L-APR: Laparoscopic abdominal perineal resection
L-TME: Laparoscopic total mesorectal excision
LARC: Locally advanced rectal cancer
MRI: Magnetic resonance imaging
NPV: Negative predictive value
NCRT: Neoadjuvant chemoradiation therapy
OS: Overall survival
pCR: Pathological complete response
PPV: Positive predictive value
TRG: Tumor regression grade

## References

[1] Kusters M, Marijnen CA, van de Velde CJ, Rutten HJ, Lahaye MJ, Kim JH, Beets-Tan RG, Beets GL (2010). Patterns of local recurrence in rectal cancer; a study of the Dutch TME trial. Eur J Surg Oncol.

[2] van Gijn W, Marijnen CA, Nagtegaal ID, Kranenbarg EM, Putter H, Wiggers T, Rutten HJ, Pahlman L, Glimelius B, van de Velde CJ, Dutch Colorectal Cancer Group (2011). Preoperative radiotherapy combined with total mesorectal excision for resectable rectal cancer: 12-year follow-up of the multicentre, randomised controlled TME trial. Lancet Oncol.

[3] Moore HG, Gittleman AE, Minsky BD, Wong D, Paty PB, Weiser M, Temple L, Saltz L, Shia J, Guillem JG (2004). Rate of pathologic complete response with increased interval between preoperative combined modality therapy and rectal cancer resection. Dis Colon Rectum.

[4] Garcia-Aguilar J, Shi Q, Thomas CR, Chan E, Cataldo P, Marcet J, Medich D, Pigazzi A, Oommen S, Posner MC (2012). A phase II trial of neoadjuvant chemoradiation and local excision for T2N0 rectal cancer: preliminary results of the ACOSOG Z6041 trial. Ann Surg Oncol.

[5] Landi F, Espin E, Rodrigues V, Vallribera F, Martinez A, Charpy C, Brunetti F, Azoulay D, de’Angelis N (2017). Pathologic response grade after long-course neoadjuvant chemoradiation does not influence morbidity in locally advanced mid-low rectal cancer resected by laparoscopy. Int J Colorectal Dis.

[6] Smith JD, Ruby JA, Goodman KA, Saltz LB, Guillem JG, Weiser MR, Temple LK, Nash GM, Paty PB (2012). Nonoperative management of rectal cancer with complete clinical response after neoadjuvant therapy. Ann Surg.

[7] Bujko K, Kolodziejczyk M, Nasierowska-Guttmejer A, Michalski W, Kepka L, Chmielik E, Wojnar A, Chwalinski M, Polish Colorectal Cancer Group (2010). Tumour regression grading in patients with residual rectal cancer after preoperative chemoradiation. Radiother Oncol.

[8] Glynne-Jones R, Wyrwicz L, Tiret E, Brown G, Rodel C, Cervantes A, Arnold D, Committee EG (2017). Rectal cancer: ESMO Clinical Practice Guidelines for diagnosis, treatment and follow-up. Ann Oncol.

[9] Boyle KM, Petty D, Chalmers AG, Quirke P, Cairns A, Finan PJ, Sagar PM, Burke D (2005). MRI assessment of the bony pelvis may help predict resectability of rectal cancer. Colorectal Dis.

[10] Killeen T, Banerjee S, Vijay V, Al-Dabbagh Z, Francis D, Warren S (2010). Magnetic resonance (MR) pelvimetry as a predictor of difficulty in laparoscopic operations for rectal cancer. Surg Endosc.

[11] Salerno G, Daniels IR, Brown G, Heald RJ, Moran BJ (2006). Magnetic resonance imaging pelvimetry in 186 patients with rectal cancer confirms an overlap in pelvic size between males and females. Colorectal Dis.

[12] de’Angelis N, Landi F, Vitali GC, Memeo R, Martinez-Perez A, Solis A, Assalino M, Vallribera F, Mercoli HA, Marescaux J, Mutter D, Ris F, Espin E (2017). Multicentre propensity score-matched analysis of laparoscopic versus open surgery for T4 rectal cancer. Surg Endosc.

[13] Nussbaum DP, Speicher PJ, Ganapathi AM, Englum BR, Keenan JE, Mantyh CR, Migaly J (2015). Laparoscopic versus open low anterior resection for rectal cancer: results from the national cancer data base. J Gastrointest Surg.

[14] Martinez-Perez A, Carra MC, Brunetti F, de’Angelis N (2017). Pathologic Outcomes of Laparoscopic vs Open Mesorectal Excision for Rectal Cancer: A Systematic Review and Meta-analysis. JAMA Surg.

[15] Fleshman J, Branda M, Sargent DJ, Boller AM, George V, Abbas M, Peters WR, Maun D, Chang G, Herline A, Fichera A, Mutch M, Wexner S (2015). Effect of Laparoscopic-Assisted Resection vs Open Resection of Stage II or III Rectal Cancer on Pathologic Outcomes: The ACOSOG Z6051 Randomized Clinical Trial. JAMA.

[16] Stevenson AR, Solomon MJ, Lumley JW, Hewett P, Clouston AD, Gebski VJ, Davies L, Wilson K, Hague W, Simes J, ALaCaRT Investigators (2015). Effect of Laparoscopic-Assisted Resection vs Open Resection on Pathological Outcomes in Rectal Cancer: The ALaCaRT Randomized Clinical Trial. JAMA.

[17] Vennix S, Pelzers L, Bouvy N, Beets GL, Pierie JP, Wiggers T, Breukink S (2014). Laparoscopic versus open total mesorectal excision for rectal cancer. Cochrane Database Syst Rev.

[18] Hoerske C, Weber K, Goehl J, Hohenberger W, Merkel S (2010). Long-term outcomes and quality of life after rectal carcinoma surgery. Br J Surg.

[19] Martinez-Perez A, Carra MC, Brunetti F, de’Angelis N (2017). Short-term clinical outcomes of laparoscopic vs open rectal excision for rectal cancer: A systematic review and meta-analysis. World J Gastroenterol.

[20] Hawkins AT, Hunt SR (2016). Watch and Wait: Is Surgery Always Necessary for Rectal Cancer?. Curr Treat Options Oncol.

[21] Lambregts DM, Rao SX, Sassen S, Martens MH, Heijnen LA, Buijsen J, Sosef M, Beets GL, Vliegen RA, Beets-Tan RG (2015). MRI and Diffusion-weighted MRI Volumetry for Identification of Complete Tumor Responders After Preoperative Chemoradiotherapy in Patients With Rectal Cancer: A Bi-institutional Validation Study. Ann Surg.

[22] Bhoday J, Smith F, Siddiqui MR, Balyasnikova S, Swift RI, Perez R, Habr-Gama A, Brown G (2016). Magnetic Resonance Tumor Regression Grade and Residual Mucosal Abnormality as Predictors for Pathological Complete Response in Rectal Cancer Postneoadjuvant Chemoradiotherapy. Dis Colon Rectum.

[23] Lambregts DM, Vandecaveye V, Barbaro B, Bakers FC, Lambrecht M, Maas M, Haustermans K, Valentini V, Beets GL, Beets-Tan RG (2011). Diffusion-weighted MRI for selection of complete responders after chemoradiation for locally advanced rectal cancer: a multicenter study. Ann Surg Oncol.

[24] Chand M, Swift RI, Tekkis PP, Chau I, Brown G (2014). Extramural venous invasion is a potential imaging predictive biomarker of neoadjuvant treatment in rectal cancer. Br J Cancer.

[25] van den Broek JJ, van der Wolf FS, Lahaye MJ, Heijnen LA, Meischl C, Heitbrink MA, Schreurs WH (2017). Accuracy of MRI in Restaging Locally Advanced Rectal Cancer After Preoperative Chemoradiation. Dis Colon Rectum.

[26] Battersby NJ, Moran B, Yu S, Tekkis P, Brown G (2014). MR imaging for rectal cancer: the role in staging the primary and response to neoadjuvant therapy. Expert Rev Gastroenterol Hepatol.

[27] Memon S, Lynch AC, Bressel M, Wise AG, Heriot AG (2015). Systematic review and meta-analysis of the accuracy of MRI and endorectal ultrasound in the restaging and response assessment of rectal cancer following neoadjuvant therapy. Colorectal Dis.

[28] Baek SJ, Kim CH, Cho MS, Bae SU, Hur H, Min BS, Baik SH, Lee KY, Kim NK (2015). Robotic surgery for rectal cancer can overcome difficulties associated with pelvic anatomy. Surg Endosc.

[29] Ferko A, Maly O, Orhalmi J, Dolejs J (2016). CT/MRI pelvimetry as a useful tool when selecting patients with rectal cancer for transanal total mesorectal excision. Surg Endosc.

[30] Salerno G, Daniels IR, Brown G, Norman AR, Moran BJ, Heald RJ (2007). Variations in pelvic dimensions do not predict the risk of circumferential resection margin (CRM) involvement in rectal cancer. World J Surg.

[31] Escal L, Nougaret S, Guiu B, Bertrand MM, de Forges H, Tetreau R, Thezenas S, Rouanet P (2018). MRI-based score to predict surgical difficulty in patients with rectal cancer. Br J Surg.

[32] Tague RG (1989). Variation in pelvic size between males and females. Am J Phys Anthropol.

[33] Boyle KM, Chalmers AG, Finan PJ, Sagar PM, Burke D (2009). Morphology of the mesorectum in patients with primary rectal cancer. Dis Colon Rectum.

[34] Fernandez Ananin S, Targarona EM, Martinez C, Pernas JC, Hernandez D, Gich I, Sancho FJ, Trias M (2014). Predicting the pathological features of the mesorectum before the laparoscopic approach to rectal cancer. Surg Endosc.

[35] Nagtegaal ID, Quirke P (2008). What is the role for the circumferential margin in the modern treatment of rectal cancer?. J Clin Oncol.

[36] Taylor FG, Quirke P, Heald RJ, Moran BJ, Blomqvist L, Swift IR, Sebag-Montefiore D, Tekkis P, Brown G, Magnetic Resonance Imaging in Rectal Cancer European Equivalence Study Study Group (2014). Preoperative magnetic resonance imaging assessment of circumferential resection margin predicts disease-free survival and local recurrence: 5-year follow-up results of the MERCURY study. J Clin Oncol.

[37] Theodoropoulos G, Wise WE, Padmanabhan A, Kerner BA, Taylor CW, Aguilar PS, Khanduja KS (2002). T-level downstaging and complete pathologic response after preoperative chemoradiation for advanced rectal cancer result in decreased recurrence and improved disease-free survival. Dis Colon Rectum.

[38] Quah HM, Chou JF, Gonen M, Shia J, Schrag D, Saltz LB, Goodman KA, Minsky BD, Wong WD, Weiser MR (2008). Pathologic stage is most prognostic of disease-free survival in locally advanced rectal cancer patients after preoperative chemoradiation. Cancer.

[39] Battersby NJ, How P, Moran B, Stelzner S, West NP, Branagan G, Strassburg J, Quirke P, Tekkis P, Pedersen BG, Gudgeon M, Heald B, Brown G (2016). Prospective Validation of a Low Rectal Cancer Magnetic Resonance Imaging Staging System and Development of a Local Recurrence Risk Stratification Model: The MERCURY II Study. Ann Surg.

[40] Kim H, Myoung S, Koom WS, Kim NK, Kim MJ, Ahn JB, Hur H, Lim JS (2016). MRI Risk Stratification for Tumor Relapse in Rectal Cancer Achieving Pathological Complete Remission after Neoadjuvant Chemoradiation Therapy and Curative Resection. PLoS One.

[41] Dresen RC, Beets GL, Rutten HJ, Engelen SM, Lahaye MJ, Vliegen RF, de Bruine AP, Kessels AG, Lammering G, Beets-Tan RG (2009). Locally advanced rectal cancer: MR imaging for restaging after neoadjuvant radiation therapy with concomitant chemotherapy. Part I. Are we able to predict tumor confined to the rectal wall?. Radiology.

[42] Dyatlov A, Gachabayov M, Bergamaschi R (2018). Nearly complete TME quality conundrum. Tech Coloproctol.

[43] Habr-Gama A, Perez RO, Sabbaga J, Nadalin W, Sao Juliao GP, Gama-Rodrigues J (2009). Increasing the rates of complete response to neoadjuvant chemoradiotherapy for distal rectal cancer: results of a prospective study using additional chemotherapy during the resting period. Dis Colon Rectum.

[44] Ahmed J, Cao H, Panteleimonitis S, Khan J, Parvaiz A (2017). Robotic vs laparoscopic rectal surgery in high-risk patients. Colorectal Dis.

[45] Jayne D, Pigazzi A, Marshall H, Croft J, Corrigan N, Copeland J, Quirke P, West N, Rautio T, Thomassen N, Tilney H, Gudgeon M, Bianchi PP (2017). Effect of Robotic-Assisted vs Conventional Laparoscopic Surgery on Risk of Conversion to Open Laparotomy Among Patients Undergoing Resection for Rectal Cancer: The ROLARR Randomized Clinical Trial. JAMA.

[46] Penna M, Hompes R, Arnold S, Wynn G, Austin R, Warusavitarne J, Moran B, Hanna GB, Mortensen NJ, Tekkis PP, TaTME Registry Collaborative (2017). Transanal Total Mesorectal Excision: International Registry Results of the First 720 Cases. Ann Surg.

[47] de’Angelis N, Portigliotti L, Azoulay D, Brunetti F (2015). Transanal total mesorectal excision for rectal cancer: a single center experience and systematic review of the literature. Langenbecks Arch Surg.

[48] Shihab OC, Taylor F, Bees N, Blake H, Jeyadevan N, Bleehen R, Blomqvist L, Creagh M, George C, Guthrie A, Massouh H, Peppercorn D, Moran BJ (2006). Diagnostic accuracy of preoperative magnetic resonance imaging in predicting curative resection of rectal cancer: prospective observational study. BMJ.

[49] Taylor FG, Quirke P, Heald RJ, Moran B, Blomqvist L, Swift I, Sebag-Montefiore DJ, Tekkis P, Brown G, MERCURY study group (2011). Preoperative high-resolution magnetic resonance imaging can identify good prognosis stage I, II, and III rectal cancer best managed by surgery alone: a prospective, multicenter, European study. Ann Surg.

[50] Edge SB, Compton CC (2010). The American Joint Committee on Cancer: the 7th edition of the AJCC cancer staging manual and the future of TNM. Ann Surg Oncol.

[51] Kapiteijn E, Marijnen CA, Nagtegaal ID, Putter H, Steup WH, Wiggers T, Rutten HJ, Pahlman L, Glimelius B, van Krieken JH, Leer JW, van de Velde CJ, Dutch Colorectal Cancer Group (2001). Preoperative radiotherapy combined with total mesorectal excision for resectable rectal cancer. N Engl J Med.

[52] Law WL, Chu KW (2004). Anterior resection for rectal cancer with mesorectal excision: a prospective evaluation of 622 patients. Ann Surg.

[53] Bianco F, Romano G, Tsarkov P, Stanojevic G, Shroyer K, Giuratrabocchetta S, Bergamaschi R, International Rectal Cancer Study G (2017). Extralevator with vs nonextralevator abdominoperineal excision for rectal cancer: the RELAPe randomized controlled trial. Colorectal Dis.

[54] Negoi I, Hostiuc S, Paun S, Negoi RI, Beuran M (2016). Extralevator vs conventional abdominoperineal resection for rectal cancer-A systematic review and meta-analysis. Am J Surg.

[55] Society of American (2018). Gastrointestinal and Endoscopic Surgeons. What is the definition of “conversion” in laparoscopic surgery among colorectal surgeons? a survey among SAGES and ASCRS. https://www.sages.org/meetings/annual-meeting/abstracts-archive/what-is-the-definition-of-conversion-in-laparoscopic-surgery-among-colorectal-surgeons-a-survey-among-sages-and-ascrs/.

[56] Dindo D, Demartines N, Clavien PA (2004). Classification of surgical complications: a new proposal with evaluation in a cohort of 6336 patients and results of a survey. Ann Surg.

[57] Rahbari NN, Weitz J, Hohenberger W, Heald RJ, Moran B, Ulrich A, Holm T, Wong WD, Tiret E, Moriya Y, Laurberg S, den Dulk M, van de Velde C (2010). Definition and grading of anastomotic leakage following anterior resection of the rectum: a proposal by the International Study Group of Rectal Cancer. Surgery.

[58] Nagtegaal ID, Marijnen CA, Kranenbarg EK, van de Velde CJ, van Krieken JH, Pathology Review C, Cooperative Clinical Investigators (2002). Circumferential margin involvement is still an important predictor of local recurrence in rectal carcinoma: not one millimeter but two millimeters is the limit. Am J Surg Pathol.

[59] Mandard AM, Dalibard F, Mandard JC, Marnay J, Henry-Amar M, Petiot JF, Roussel A, Jacob JH, Segol P, Samama G, Ollivier JM, Bonvalot S, Gignoux M (1994). Pathologic assessment of tumor regression after preoperative chemoradiotherapy of esophageal carcinoma. Clinicopathologic correlations. Cancer.

[60] Smith KD, Tan D, Das P, Chang GJ, Kattepogu K, Feig BW, Skibber JM, Rodriguez-Bigas MA (2010). Clinical significance of acellular mucin in rectal adenocarcinoma patients with a pathologic complete response to preoperative chemoradiation. Ann Surg.

[61] Ogiso S, Yamaguchi T, Hata H, Fukuda M, Ikai I, Yamato T, Sakai Y (2011). Evaluation of factors affecting the difficulty of laparoscopic anterior resection for rectal cancer: “narrow pelvis” is not a contraindication. Surg Endosc.

[62] Smith NJ, Barbachano Y, Norman AR, Swift RI, Abulafi AM, Brown G (2008). Prognostic significance of magnetic resonance imaging-detected extramural vascular invasion in rectal cancer. Br J Surg.

[63] Shihab OC, Taylor F, Salerno G, Heald RJ, Quirke P, Moran BJ, Brown G (2011). MRI predictive factors for long-term outcomes of low rectal tumours. Ann Surg Oncol.

[64] Vecchio FM, Valentini V, Minsky BD, Padula GD, Venkatraman ES, Balducci M, Micciche F, Ricci R, Morganti AG, Gambacorta MA, Maurizi F, Coco C (2005). The relationship of pathologic tumor regression grade (TRG) and outcomes after preoperative therapy in rectal cancer. Int J Radiat Oncol Biol Phys.

[65] Akiyoshi T, Kuroyanagi H, Oya M, Konishi T, Fukuda M, Fujimoto Y, Ueno M, Miyata S, Yamaguchi T (2009). Factors affecting the difficulty of laparoscopic total mesorectal excision with double stapling technique anastomosis for low rectal cancer. Surgery.

[66] Targarona EM, Balague C, Pernas JC, Martinez C, Berindoague R, Gich I, Trias M (2008). Can we predict immediate outcome after laparoscopic rectal surgery? Multivariate analysis of clinical, anatomic, and pathologic features after 3-dimensional reconstruction of the pelvic anatomy. Ann Surg.

